# Validity of mortality risk prediction scores in critically ill patients with secondary pulmonary embolism

**DOI:** 10.17305/bb.2024.10202

**Published:** 2024-08-01

**Authors:** Martin J Ryll, Toby N Weingarten, Darrell R Schroeder, Juraj Sprung

**Affiliations:** 1 Faculty of Medicine, Ludwig Maximilian University of Munich, Munich, Germany; 2 Department of Anesthesiology and Perioperative Medicine, Mayo Clinic College of Medicine and Science, Rochester, MN, USA; 3 Health Sciences Research, Division of Epidemiology, Mayo Clinic College of Medicine and Science, Rochester, MN, USA

**Keywords:** Acute Physiology and Chronic Health Evaluation-IV (APACHE-IV), critical care, ICU-sPESI, intensive care unit (ICU), mortality, Pulmonary Embolism Severity Index (PESI), pulmonary embolism (PE), simplified PESI (sPESI)

## Abstract

Pulmonary embolism (PE) is a feared complication in the ICU, significantly impacting morbidity and mortality of the patients affected. Herein, we assess the use of the Acute Physiology and Chronic Health Evaluation-IV (APACHE-IV) and PE-specific risk scores to predict mortality among intensive care unit (ICU) patients who developed secondary PE. This retrospective cohort study is using information from 208 United States critical care units recorded in the eICU Collaborative Research Database during 2014 and 2015. We calculated APACHE-IV, Pulmonary Embolism Severity Index (PESI), simplified PESI (sPESI), and ICU-sPESI scores and compared their predicting performance using the area under the receiver operating characteristic (AUROC) curve. Of 812 patients included in our study, 150 died (mortality, 18.5% [95% CI, 15.8%–21.1%]). Compared to survivors, non-survivors had higher APACHE-IV (86 vs 52, *P* < 0.001), PESI (170 vs 129, *P* < 0.001), sPESI (2 vs 2, *P* < 0.001), and ICU-sPESI (4 vs 2, *P* < 0.001) scores. AUROCs were 0.790 (APACHE-IV); 0.737 (PESI); 0.726 (ICU-sPESI); and 0.620 (sPESI). APACHE-IV performed significantly better than all three PE-specific mortality scores (APACHE-IV vs PESI, *P* ═ 0.041; APACHE-IV vs sPESI, *P* ═ 0.001; and APACHE-IV vs ICU-sPESI, *P* ═ 0.021). Both the PESI and ICU-sPESI outperformed the sPESI (PESI vs sPESI, *P* ═ 0.001; ICU-sPESI vs sPESI, *P* < 0.001). APACHE-IV score was found to be the best instrument for predicting mortality risk, but PESI and ICU-sPESI scores may be used when APACHE-IV is unavailable. sPESI AUROC suggests the absence of a sufficient discriminative value to be used as a predictor of mortality in patients with secondary PE.

## Introduction

Depending on the clinical presentation, mortality after pulmonary embolism (PE) may vary widely [1–4]. Specifically, in the absence of right ventricular dysfunction, mortality in patients with PE as a primary diagnosis on admission (primary PE) may be as low as 1% but may exceed 50% when PE presents with hemodynamic instability or shock [4, 5]. When PE occurs during a patient’s stay in the intensive care unit (ICU) (secondary PE), mortality is likely to be high. The best-known all-cause mortality prediction instrument for critically ill patients admitted to the ICU is the Acute Physiology and Chronic Health Evaluation-IV (APACHE-IV). However, the practical use of APACHE-IV is complex, as it requires gathering 129 variables to calculate the score [6, 7]. Several disease-specific scoring systems have been developed to predict mortality for patients with primary PE [2, 8–10]. Two commonly used systems are the Pulmonary Embolism Severity Index (PESI) [2] and the simplified PESI score (sPESI) [9]. We recently proposed a modified sPESI score (ICU-sPESI) by adding three equally weighted binary variables that denote the severity of PE on presentation: 1) intubation status; 2) altered mental status; and 3) use of vasopressors [10]. Across the nine ICU-sPESI points, we identified four classes that provide good discrimination of the mortality risk [10]. Clinical components used to construct PESI, sPESI, and ICU-sPESI are detailed in our earlier report [10]. It is not known whether PE-specific scores, designed for primary PE, could be used as predictive mortality tools in patients with secondary PE.

Our study includes patients admitted to the ICU with critical illness and a subsequent diagnosis of PE. In this study, we compared the mortality prediction abilities of the APACHE-IV, PESI, sPESI, and ICU-sPESI to assess their accuracy in patients with secondary PE. We hypothesized that in this complex clinical scenario, PE-specific scores would be a poor predictor of mortality compared to their use after primary PE. We also hypothesized that, given the complexity of primary disease complicated with PE, APACHE-IV would predict all-cause mortality better than PE-specific scores.

## Materials and methods

### Patient selection, inclusion, and exclusion criteria

We included all patients who developed PE within 48 h of ICU admission. In the eICU-CRD, a running active list of diagnoses, including timestamps, is recorded in the “diagnosis” table, which we used to identify the time of PE diagnosis in regard to ICU admission. We excluded any patients with missing key data (e.g., sex, APACHE-IV scores, survival outcome, and primary admission diagnosis). We also excluded all patients with primary admission diagnoses suggestive of primary PE (e.g., thrombus, arterial; thrombectomy; embolectomy; thrombosis, vascular [deep vein]; chest pain, atypical [noncardiac chest pain]; chest pain, respiratory; vena cava filter insertion).

### Calculation of mortality scores

APACHE-IV scores are calculated and reported in the eICU-CRD. Variables included in the calculation of PE mortality scores were detailed in our earlier report [10]. Briefly, to calculate the PESI score, different characteristics are each assigned a number of points as follows: 10 points ═ male sex, history of heart failure, history of chronic lung disease; 20 points ═ heart rate ≥110/min, respiratory rate ≥30/min, temperature <36 ^∘^C, oxyhemoglobin saturation <90%; 30 points ═ history of cancer, systolic blood pressure <100 mmHg; 60 points ═ altered mental status; in addition, one point was added for each year of age (e.g., 75 years ═ 75 points). To calculate the sPESI score, the presence of the following characteristics was assigned 1 score point each (maximum score of 6 points): age >80 years, history of cancer, chronic cardiopulmonary disease, heart rate ≥110/min, systolic blood pressure <100 mmHg, oxyhemoglobin saturation <90%. Finally, for ICU-sPESI score calculation, in addition to the original 6 sPESI points, three additional binary characteristics were included (maximum score of 9 points): intubation status; altered mental status; and the use of vasoactive drug infusions [10]. Across the 9 ICU-sPESI points, we identified four classes that provided excellent discrimination of the mortality risk: ≤2 points ≜ Class I (low risk); 3–4 points ≜ Class II (intermediate risk); 5–6 points ≜ Class III (high risk); ≥7 points ≜ Class IV (very high risk) [10].

### Data source

The data for this study were extracted from the eICU Collaborative Research Database version 2.0 (eICU-CRD). This extensive critical care database, developed by Philips Healthcare in partnership with the Massachusetts Institute of Technology Laboratory for Computational Physiology, contains diverse clinical information from over 200,000 ICU admissions in 208 hospitals throughout the United States, covering the years 2014 and 2015 [11, 12].

### Ethical statement

This study was conducted in accordance with the Declaration of Helsinki and the Strengthening the Reporting of Observational Studies in Epidemiology (STROBE) reporting guidelines [13]. Due to its retrospective design, the absence of direct patient involvement, and a security framework meeting the Safe Harbor criteria for re-identification risk (as verified by Privacert in Cambridge, MA, USA; Health Insurance Portability and Accountability Act Certification no. 1031219-2), the study did not require institutional review board approval. Data extraction and analysis were managed by Martin J. Ryll, who obtained certified access (CITI ID 50703292).

### Statistical analysis

As previously described [10], vital signs were assessed from medians calculated over 30 min and we used the most aberrant values for the final computation of scores. Continuous variables were described using the median and interquartile range (IQR), while categorical and binary variables were summarized by frequency and percentage. The comparison between surviving and non-surviving groups for categorical and binary variables was conducted using the chi-square test or Fisher’s Exact test, as appropriate. The Shapiro–Wilk test was applied to assess the normality of continuous variables, which were then compared using the independent *t*-test or Mann–Whitney *U* test.

Mortality predictions for each score were derived using a univariate logistic regression model with the score as the independent and in-hospital mortality as the dependent variable. Area under the receiver operating characteristic (AUROC) curves were calculated for the four scores and the AUROCs were compared as described by Hanley and McNeil [14]. Of note, an AUROC of 0.5 suggests no discrimination (i.e., ability to reliably predict an outcome based on score values), and is deemed as acceptable between 0.7 and 0.8, as excellent from 0.8–0.9, and as outstanding above 0.9 [15]. To illustrate the fit of our models across various scoring intervals, we plotted both the predicted and actual in-hospital mortality rates against the deciles for the APACHE-IV and PESI scores, as well as against the actual score values for the sPESI and ICU-sPESI scores. Survival curves for different PESI categories, sPESI scores, and ICU-sPESI categories were generated using the Kaplan–Meier method, with all observations being censored 55 days following ICU admission. To evaluate the differences between these survival curves, we initially applied a multivariate log-rank test, followed by pairwise log-rank tests (*P* values adjusted using the Bonferroni correction) upon identifying any differences. All tests conducted were two-sided, considering *P* values <0.05 statistically significant. Python v.3.9 (Python Software Foundation, Wilmington, Delaware, USA) with its libraries Pandas (v.1.4.3) [16], NumPy (v.1.23.0) [17], SciPy (v.1.10.1) [18], scikit-learn (v.1.10.1) [19], Statsmodels (v.0.13.5) [20], and lifelines (v.0.27.7) [21] was used for statistical analysis, while Matplotlib (v.3.7.1) [22] and Seaborn (v.0.12.0) were used for visualization [23].

## Results

A total of 1028 patients admitted to the ICU were subsequently diagnosed with secondary PE within 48 h of admission. We excluded 216 cases due to missing data, resulting in a cohort of 812 patients with secondary PE. Of these, 150 did not survive hospital discharge, resulting in a mortality rate of 18.5% (95% CI, 15.8%–21.1%). The median time to all-cause death was 6.2 (IQR 1.7, 11.7) days.

Table 1 and Table S1 summarize demographic and comorbid variables between survivors and non-survivors. Compared to survivors, non-survivors were older (median age, 70 vs 65 years; *P* ═ 0.002) and had higher rates of cancer history (30.0% vs 21.1%; *P* ═ 0.026). Table 2 provides a detailed overview of the primary ICU admission diagnoses.

**Table 1:** Demographics and comorbidities among patients admitted to the ICU with a secondary PE

**Characteristics**	**All patients** **(*N* ═ 812)**	**Survivors** **(*n* ═ 662)**	**Non-survivors** **(*n* ═ 150)**	***P* value**
Age, years	66 [53–76]	65 [53–75]	70 [59–78]	0.002
*Cardiovascular comorbidities*				
Hypertension	411 (50.6)	335 (50.6)	76 (50.7)	>0.99
Prior venous thrombosis/PE	198 (24.4)	165 (24.9)	33 (22.0)	0.517
Coronary artery disease^a^	173 (21.3)	136 (20.5)	37 (24.7)	0.316
Congestive heart failure	137 (16.9)	113 (17.1)	24 (16.0)	0.845
Cardiac dysrhythmia^b^	118 (14.5)	93 (14.0)	25 (16.7)	0.488
Pulmonary conditions^c^	232 (28.6)	198 (29.9)	34 (22.7)	0.094
Neurologic conditions^d^	81 (10.0)	68 (10.3)	13 (8.7)	0.659
Cancer	185 (22.8)	140 (21.1)	45 (30.0)	0.026
*Other comorbidities*				
Diabetes mellitus	193 (23.8)	157 (23.7)	36 (24.0)	>0.99
Liver cirrhosis	19 (2.3)	13 (2.0)	6 (4.0)	0.234
Renal insufficiency	53 (6.5)	41 (6.2)	12 (8.0)	0.531

Data are count (percentage) or median [IQR]. ^a^Includes other major vessel atherosclerotic disease. ^b^Includes patients with pacemakers and/or automatic implantable cardioverter-defibrillators. ^c^Includes patients with obstructive and restrictive lung disease, and patients with home-oxygen use. ^d^Includes patients with seizures, dementia, intracranial masses, and neuromuscular disease. PE: Pulmonary embolism; ICU: Intensive care unit; IQR: Interquartile range.

**Table 2:** Primary ICU-admission diagnoses among critically ill patients with a secondary PE

**Primary diagnoses**	**All patients** **(*N* ═ 812)**	**Survivors** **(*n* ═ 662)**	**Non-survivors** **(*n* ═ 150)**
*Postsurgical*			
Gastrointestinal	24 (3.0)	19 (2.9)	5 (3.3)
Neurologic	10 (1.2)	8 (1.2)	2 (1.3)
Cardiovascular	9 (1.1)	7 (1.1)	2 (1.3)
Respiratory	8 (1.0)	7 (1.1)	1 (0.7)
Other	14 (1.7)	12 (1.8)	2 (1.3)
Sepsis	192 (23.6)	158 (23.9)	34 (22.7)
*Cardiovascular*			
MI/cardiac arrest/angina	74 (9.1)	45 (6.8)	29 (19.3)
Arrythmia	50 (6.2)	43 (6.5)	7 (4.7)
Congestive heart failure	40 (4.9)	36 (5.4)	4 (2.7)
Other	54 (6.7)	48 (7.3)	6 (4.0)
*Respiratory*			
Pneumonia	59 (7.3)	45 (6.8)	14 (9.3)
Emphysema/bronchitis	26 (3.2)	25 (3.8)	1 (0.7)
Respiratory arrest	22 (2.7)	16 (2.4)	6 (4.0)
ARDS	10 (1.2)	8 (1.2)	2 (1.3)
Other	67 (8.3)	50 (7.6)	17 (11.3)
*Gastrointestinal*			
GI bleeding	33 (4.1)	32 (4.8)	1 (0.7)
Other	15 (1.8)	11 (1.7)	4 (2.7)
*Neurologic*			
Stroke/ICB	29 (3.6)	23 (3.5)	6 (4.0)
Other	30 (3.7)	28 (4.2)	2 (1.3)
Endocrine	8 (1.0)	7 (1.1)	1 (0.7)
Genitourinary	12 (1.5)	11 (1.7)	1 (0.7)
Hematologic	16 (2.0)	15 (2.3)	1 (0.7)
MSK/skin/trauma	10 (1.2)	8 (1.2)	2 (1.3)

Data are count (percentage). ARDS: Acute respiratory distress syndrome; GI: Gastrointestinal; ICB: Intracranial bleed; ICU: Intensive care unit; MI: Myocardial infarction; MSK: Musculoskeletal; PE: Pulmonary embolism.

APACHE-IV, PESI, sPESI, ICU-sPESI scores, and PESI/ICU-sPESI risk classes, as well as their distribution, are included in Table 3. Compared to survivors, non-survivors had higher APACHE-IV (86 vs 52; *P* < 0.001), PESI (170 vs 129; *P* < 0.001), sPESI (2 vs 2; *P* < 0.001), and ICU-sPESI (4 vs 2; *P* < 0.001) scores.

**Table 3:** APACHE-IV, PESI, sPESI, and ICU-sPESI classes and scores

**Scores**	**All patients** **(*N* ═ 812)**	**Survivors** **(*n* ═ 662)**	**Non-survivors** **(*n* ═ 150)**	**Actual mortality**	***P* value**
APACHE-IV scores	56.0 [43.0–78.0]	52.0 [40.0–68.0]	86.0 [60.2–119.5]	–	<0.001
PESI score	137.0 [97.0–172.2]	129.0 [90.0–163.0]	170.0 [141.0–206.8]	–	<0.001
PESI risk class^a^					<0.001
I	58 (7.1)	56 (8.5)	2 (1.3)	3.4%	
II	89 (11.0)	86 (13.0)	3 (2.0)	3.4%	
III	90 (11.1)	86 (13.0)	4 (2.7)	4.4%	
IV	112 (13.8)	95 (14.4)	17 (11.3)	15.2%	
V	463 (57.0)	339 (51.2)	124 (82.7)	26.8%	
sPESI score	2.0 [1.0–3.0]	2.0 [1.0–3.0]	2.0 [1.0–3.0]	–	<0.001
sPESI points^b^				–	<0.001
0	111 (13.7)	101 (15.3)	10 (6.7)	9.0%	
1	219 (27.0)	186 (28.1)	33 (22.0)	15.1%	
2	242 (29.8)	200 (30.2)	42 (28.0)	17.4%	
3	176 (21.7)	137 (20.7)	39 (26.0)	22.2%	
4	56 (6.9)	34 (5.1)	22 (14.7)	39.3%	
5	8 (1.0)	4 (0.6)	4 (2.7)	50.0%	
ICU-sPESI score	3.0 [1.0–4.0]	2.0 [1.0–4.0]	4.0 [3.0–5.0]	–	<0.001
ICU-sPESI risk class^c^					<0.001
I	367 (45.2)	336 (50.8)	31 (20.7)	8.4%	
II	303 (37.3)	245 (37.0)	58 (38.7)	19.1%	
III	127 (15.6)	77 (11.6)	50 (33.3)	39.4%	
IV	15 (1.8)	4 (0.6)	11 (7.3)	73.3%	

Data are count (percentage) or median [IQR]. ^a^PESI score classes: Class I, ≤65 (very low risk); Class II, 66–85 (low risk); Class III, 86–105 (intermediate risk); Class IV, 106–125 (high risk); Class V, >125 (very high risk). Generally, Class I and II are considered low-risk; Classes III-V, high risk. ^b^sPESI score is generally considered low risk if 0, and high risk if ≥1. No patient in this cohort had a score of 6 points. ^c^ICU-sPESI classes: Class I ═ ≤2 points (low risk); Class II ═ 3–4 points (intermediate risk); Class III ═ 5–6 points (high risk); Class IV ═ ≥7 points (very high risk). APACHE-IV: Acute Physiology and Chronic Health Evaluation IV; ICU-sPESI: ICU-modified Simplified Pulmonary Embolism Severity Index; PESI: Pulmonary Embolism Severity Index; sPESI: Simplified Pulmonary Embolism Severity Index.

Table S2 gives an overview of PE-specific score components for survivors and non-survivors. While the continuous variable for age (PESI) differed between survivors and non-survivors, age greater than 80 (sPESI, ICU-sPESI) did not. Furthermore, rates of male sex (PESI), a history of chronic lung disease (PESI), heart failure (PESI), or chronic cardiopulmonary disease (sPESI, ICU-sPESI) did not differ significantly between survivors and non-survivors. All vital signs and treatment variables were associated with mortality (all *P* ≤ 0.001, except for heart rate [*P* ═ 0.045]).

**Figure 1.**
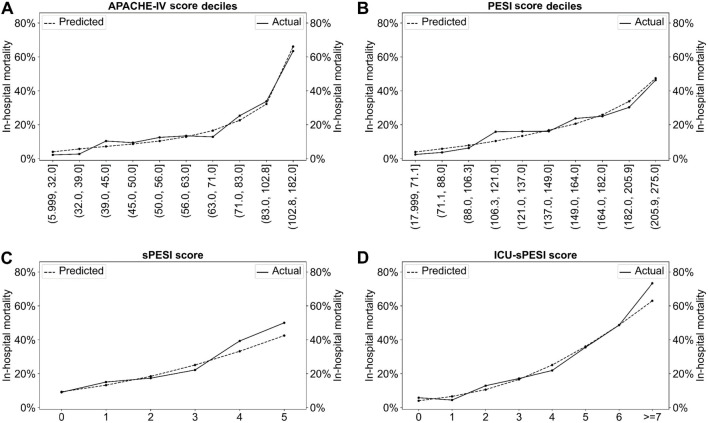
**Actual vs predicted mortality for (A) APACHE-IV, (B) PESI (scores shown in deciles), (C) sPESI, and (D) ICU-sPESI (scores shown in direct score values).** APACHE-IV: Acute Physiology and Chronic Health Evaluation IV; ICU-sPESI: ICU-modified Simplified Pulmonary Embolism Severity Index; PESI: Pulmonary Embolism Severity Index; sPESI: Simplified Pulmonary Embolism Severity Index.

**Figure 2.**
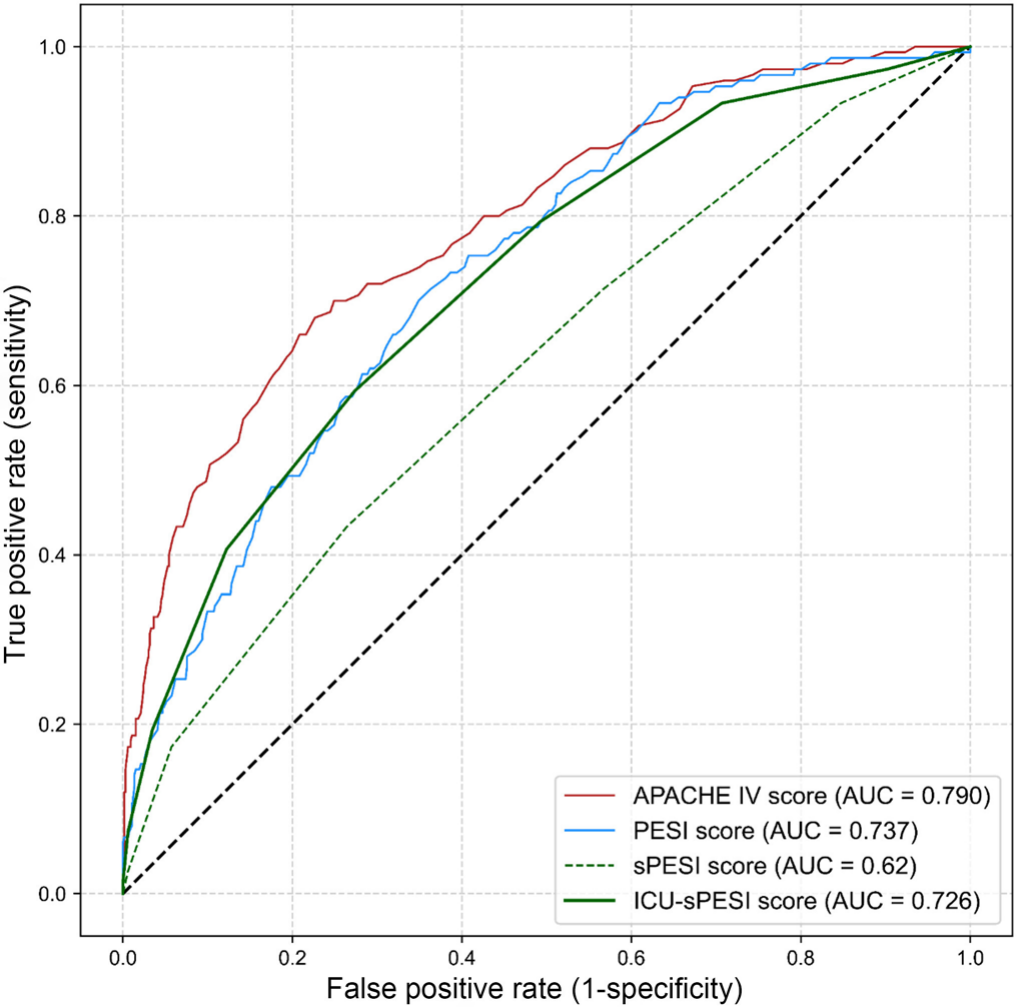
**Receiver operating characteristic curves for the APACHE-IV, PESI, sPESI, and ICU-sPESI for predicting all-cause mortality in ICU patients with secondary PE.** APACHE-IV: Acute Physiology and Chronic Health Evaluation IV; ICU-sPESI: ICU-modified Simplified Pulmonary Embolism Severity Index; PESI: Pulmonary Embolism Severity Index; sPESI: Simplified Pulmonary Embolism Severity Index; ICU: Intensive care unit; PE: Pulmonary embolism.

Figure 1 shows the observed and predicted in-hospital mortality from each of the univariate logistic regression models for APACHE-IV, PESI, sPESI, and ICU-sPESI. All four scores demonstrated a comparable concordance of observed and predicted in-hospital mortality across their respective score ranges, illustrating accurate calibration for the logistic regression models. Receiver operating characteristic curves were used to assess the discriminative ability of the scores regarding mortality prediction. The AUROC for APACHE-IV was 0.790; for PESI, 0.737; for ICU-sPESI, 0.726; and, finally, for sPESI, 0.620 (Figure 2). APACHE-IV performed significantly better than the PE-specific mortality scores (APACHE-IV vs PESI, *P* ═ 0.041; APACHE-IV vs sPESI, *P* ═ 0.001; APACHE-IV vs ICU-sPESI, *P* ═ 0.021). Both PESI and ICU-sPESI outperformed sPESI (PESI vs sPESI, *P* ═ 0.001; ICU-sPESI vs sPESI, *P* < 0.001), but PESI and ICU-sPESI had comparable performances (PESI vs ICU-sPESI, *P* ═ 0.605).

**Figure 3.**
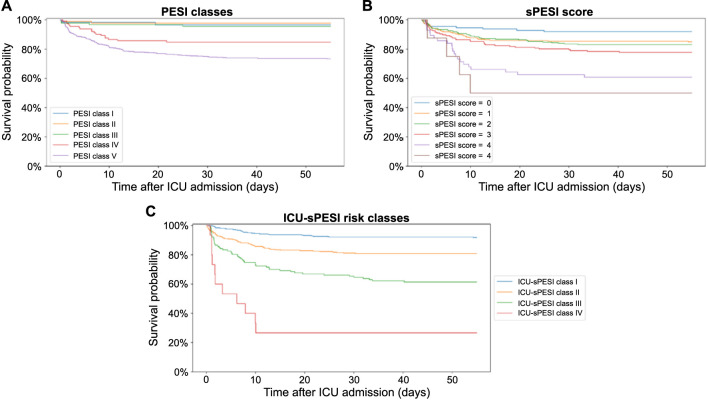
**Kaplan–Meier survival curves for ICU patients with secondary PE shown for PESI classes, sPESI scores, and ICU-sPESI classes.** For all scores and classes, there was a significant difference in survival across all groups (multivariate log-rank test; *P* < 0.001). The following pairwise survival curve comparisons differed significantly (log-rank test, adjusted for multiple comparisons by Bonferroni correction). (A) PESI classes: PESI Class V vs III, II, and I: All *P* values ≤ 0.002. PESI Class IV vs II: *P ═* 0.021. (B) sPESI score: sPESI score 5 vs 0 and 4 vs 2, 1, 0: All *P* values ≤ 0.001. sPESI score 3 vs 0: *P ═* 0.036. (C) ICU-sPESI classes: All comparisons with *P* values <0.001 except for ICU-sPESI Class IV vs III with a *P* ═ 0.005. All other comparisons were not significant. APACHE-IV: Acute Physiology and Chronic Health Evaluation IV; ICU-sPESI: ICU-modified Simplified Pulmonary Embolism Severity Index; PESI: Pulmonary Embolism Severity Index; sPESI: Simplified Pulmonary Embolism Severity Index, ICU: Intensive care unit; PE: Pulmonary embolism.

Figure 3 shows the Kaplan–Meier survival curves for PESI classes, sPESI scores, and ICU-sPESI classes. For all scores, survival differed across groups and decreased as the scores increased (multivariate log-rank test, *P* < 0.001). Individual comparisons of PESI classes and sPESI scores, adjusted for multiple comparisons, showed few differences in survival curves, almost exclusively for PESI Class V and sPESI score 4. ICU-sPESI classes demonstrated significantly different survival for all individual comparisons (*P* < 0.001 for all individual comparisons, except for Class III vs IV [*P* ═ 0.005]). Details regarding all Bonferroni-corrected pairwise comparisons between respective scores and risk classes are provided in the footnote of Figure 3.

## Discussion

In our cohort of critically ill patients with secondary PE, in-hospital all-cause mortality was 18.5%. APACHE-IV demonstrated a better discriminatory ability to predict mortality than the PESI or ICU-sPESI. However, despite being inferior to the APACHE-IV, the performances of PESI and ICU-sPESI were still within an acceptable range [15]. In contrast, the sPESI should not be considered a useful predictive tool in patients with secondary PE.

Using the eICU Collaborative Research Database, we recently reported a 6.3% mortality rate after primary PE [10], and using the same database in our current study, we found a 3-fold higher mortality rate for patients with secondary PE. This mortality rate reflects the effects of primary critical illness compounded with secondary PE. Similarly, a study by Miró et al. [24] reported higher mortality in patients with secondary compared to primary PE (12.8% vs 5.3%). In another study, critically ill patients who developed secondary PE had a mortality rate of 20% [25], similar to that reported in our current study.

Given its high level of accuracy, the APACHE-IV has proven superior to other prognosticating scores in predicting all-cause mortality in critically ill patients [26]. We demonstrated that APACHE-IV performed better than the PE-specific scores when used to predict outcomes in critically ill patients with secondary PE. This is likely because APACHE-IV is calculated from a very large number of clinical variables, which accounts for a wide range of health alterations not captured by the variables in PE-specific scales [26]. However, APACHE-IV’s implementation is far more complex than that of the PESI and ICU-sPESI and requires specialized training of staff [27]. For this reason, we conducted this study to examine whether less complex mortality-risk scores, such as PESI, ICU-sPESI, or sPESI, could replace APACHE-IV in clinical settings where PE occurs in addition to, and potentially exacerbates, an underlying critical illness.

Furthermore, we demonstrated that the predictive performance of PE-specific mortality-risk scores in patients with secondary PE was lower than in patients with primary PE [10]. Specifically, for patients with primary PE the APACHE-IV, PESI, ICU-sPESI, and sPESI AUROCs were 0.870, 0.848, 0.847, and 0.777 [10], and after secondary PE 0.790, 0.737, 0.726, and 0.620, respectively. This difference may be attributed to the fact that the PE-specific scores are designed to capture specific PE-associated symptomatology. In other words, the presence of underlying complex critical illness may confound the “typical” mortality-risk predictors for which these PE-specific scores were designed, leading to the observed lower prognosticating ability in these patients.

One of the main limitations of this study is its retrospective nature. The disease acuity for which a patient is admitted to the ICU as well as PE severity may vary, and the retrospective inclusion of signs and symptoms may not objectively capture the clinical status, which may in turn lead to overestimation or underestimation of the outcome by the scoring systems. Furthermore, the eICU-CRD does not report imaging data, such as computerized tomography scans or echocardiographic data, prohibiting severity assessment via these measurements. As a result, the eICU does not specify how PE is confirmed, resulting in a slight risk of misdiagnosis. Our results are based on observations from a multicenter database comprising more than 200 hospitals in the United States, which provides good generalizability to industrialized countries. However, our results cannot be generalized to geographic areas that may have different admission strategies, guidelines for treatment, protocols, definitions of cut-off values, or substandard treatments. Finally, the number of patients in the highest-risk scores/classes for sPESI and ICU-sPESI was relatively small, which may limit the accuracy of high-mortality risk estimates.

## Conclusion

The mortality rate of patients diagnosed with PE after ICU admission is high. The APACHE-IV is the best mortality predictor for these patients; however, AUROCs for PESI and ICU-sPESI were still within a range commonly considered acceptable, while the sPESI lacks sufficient discriminative value as a predictor of mortality in patients with secondary PE.

## Supplemental data

**Table S1:** PESI, sPESI, and ICU-sPESI score components

**Score components**	**All patients** **(*N* ═ 812)**	**Survivors** **(*n* ═ 662)**	**Non-survivors** **(*n* ═ 150)**	***P* values ^e^**
*Demographics and comorbidities*				
Age, years ^a^	66 [53–76.0]	65 [53–75]	70 [59–79]	0.002
Age >80 years ^b^	118 (14.5)	93 (14.0)	25 (16.7)	0.488
Sex (male) ^a^	411 (50.6)	325 (49.1)	86 (57.3)	0.083
Chronic lung disease ^a^	232 (28.6)	198 (29.9)	34 (22.7)	0.094
Heart failure ^a^	137 (16.9)	113 (17.1)	24 (16.0)	0.845
Chronic CP disease ^b^	347 (42.7)	287 (43.4)	60 (40.0)	0.510
History of cancer ^a,b^	185 (22.8)	140 (21.1)	45 (30.0)	0.026
*Vitals and treatments*				
Heart rate ≥110 ^a,b^	271 (33.4)	210 (31.7)	61 (40.7)	0.045
SBP <100 mmHg ^a,b^	440 (54.2)	337 (50.9)	103 (68.7)	<0.001
Temperature <36.0 ^∘^C ^a^	65 (8.0)	34 (5.1)	31 (20.7)	<0.001
Respiratory rate ≥30 ^a^	187 (23.0)	137 (20.7)	50 (33.3)	0.001
SaO2 % <90 ^a,b^	134 (16.5)	86 (13.0)	48 (32.0)	<0.001
Altered mental status ^a,c^	328 (40.4)	229 (34.6)	99 (66.0)	<0.001
Intubated ^c^	296 (36.5)	203 (30.7)	93 (62.0)	<0.001
Vasopressors/inotropes ^c,d^	158 (19.5)	95 (14.4)	63 (42.0)	<0.001

Data are count (percentage) or median [IQR]. Vital signs and treatments are obtained within 12 h before and after secondary PE diagnosis. ^a^ Included in the PESI. ^b^ Included in the sPESI. ^c^ Included in the ICU-sPESI. ^d^ Include vasoconstrictors and or inotropic infusions. ^e^ P values were not adjusted for multiple comparisons. SBP: Systolic blood pressure; CP: Cardiopulmonary; SaO2%: Percentage of oxyhemoglobin saturation; ^∘^C: Degrees Celsius; APACHE-IV: Acute Physiology and Chronic Health Evaluation IV; PESI: Pulmonary Embolism Severity Index; sPESI: Simplified PESI; ICU-sPESI: ICU-modified sPESI.

**Table S2:** Additional details of comorbidities prior to ICU admission of patients with PE who required ICU admission

**Comorbidities**	**All patients** **(*N* ═ 812)**	**Survivors** **(*n* ═ 662)**	**Non-survivors** **(*n* ═ 150)**	***P* values**
COPD^a^				0.250
Mild	40 (4.9)	37 (5.6)	3 (2.0)	
Moderate	86 (10.6)	72 (10.9)	14 (9.3)	
Severe	45 (5.5)	35 (5.3)	10 (6.7)	
Cardiac dysrhythmias				0.290
Atrial fibrillation	91 (11.2)	70 (10.6)	21 (14.0)	
Other	7 (0.9)	5 (0.8)	2 (1.3)	
Cancer history				0.020
Genitourinary	31 (3.8)	26 (3.9)	5 (3.3)	
GI and hepatobiliary	49 (6.0)	32 (4.8)	17 (11.3)	
Respiratory	39 (4.8)	27 (4.1)	12 (8.0)	
Breast	28 (3.4)	25 (3.8)	3 (2.0)	
Other	37 (4.6)	29 (4.4)	8 (5.3)	
Diabetes mellitus				0.273
No treatment	24 (3.0)	17 (2.6)	7 (4.7)	
Antidiabetic medications only	95 (11.7)	82 (12.4)	13 (8.7)	
Insulin dependent	74 (9.1)	58 (8.8)	16 (10.7)	
Renal failure				0.282
Without dialysis	21 (2.6)	20 (3.0)	1 (0.7)	
With dialysis	24 (3.0)	20 (3.0)	4 (2.7)	
Prior PE				0.069
Single	159 (19.6)	139 (21.0)	20 (13.3)	
Multiple	6 (0.7)	5 (0.8)	1 (0.7)	

Data are count (percentage). Individual features had no missing values. ^a^ Reported as “mild”, “moderate”, or “severe” in the eICU database. COPD: Chronic obstructive pulmonary disease; GI: Gastrointestinal; ICU: Intensive care unit; PE: Pulmonary embolism.

## Data Availability

All code for data extraction and analysis is documented online at https://github.com/RyllMartin/eICU_ICU_sPESI_validation_secondary_PE.
